# Characteristics of different risk factors and fasting plasma glucose for identifying GDM when using IADPSG criteria: a cross-sectional study

**DOI:** 10.1186/s12884-018-1875-1

**Published:** 2018-06-13

**Authors:** Maryam Saeedi, Ulf Hanson, David Simmons, Helena Fadl

**Affiliations:** 1 Örebro University hospital, Örebro University, Örebro, Sweden; 2 Department of Women’s and Children’s health, Uppsala University, Uppsala, Sweden; 3 Department of Obstetrics and Gynecology, School of medical health and sciences, Örebro University Hospital, Örebro, Sweden; 4 Macarthur Clinical School, Western Sydney University, Campbelltown, Australia

**Keywords:** Gestational diabetes mellitus, Screening, Fasting plasma glucose, Risk factors, Sensitivity

## Abstract

**Background:**

The Swedish National Board of Health and Welfare (SNBHW) recommended the new diagnostic criteria for GDM based upon Hyperglycaemia and Adverse Pregnancy Outcomes (HAPO) study thresholds. Due to limited knowledge base, no recommendations were made on GDM screening. The aim of this study is to evaluate test characteristics of risk factors and fasting blood glucose as screening tests for diagnosing GDM using diagnostic thresholds based upon HAPO study 1.75/2.0 (model I/II respectively) odds ratio for adverse pregnancy outcomes.

**Methods:**

This cross-sectional, population-based study included all pregnant women who attended maternal health care in Örebro County, Sweden between the years 1994–96. A 75 g OGTT with capillary fasting and 2-h blood glucose was offered to all pregnant women at week 28–32. Risk factors and repeated random glucose samples were collected. Sensitivity, specificity and predictive values of blood glucose were calculated.

**Results:**

Prevalence of GDM was 11.7% with model I and 7.2% with the model II criteria. Risk factors showed 28%, (95% CI 24–32) and 31%, (95% CI 25–37) sensitivity for model I and II respectively. A fasting cut off ≥4.8 mmol/l occurred in 24% of women with 91%, (95% CI 88–94) sensitivity and 85%, (95% CI 83–86) specificity using model I while a fasting cut off ≥5.0 mmol/l occurred in 14% with 91%, (95% CI 87–94) sensitivity and 92%, (95% CI 91–93) specificity using model II.

**Conclusion:**

Risk factor screening for GDM was found to be poorly predictive of GDM but fasting glucose of 4.8–5.0 mmol/l showed good test characteristics irrespective of diagnostic model and results in a low rate of OGTTs.

## Background

The prevalence of gestational diabetes mellitus (GDM) varies from 1 to 28% [1, 2] mainly depending on screening and diagnostic criteria, the population’s ethnic composition and prevalence of type 2 diabetes (T2DM) [3]. The International Association of Diabetes and Pregnancy Study Groups (IADPSG) diagnostic criteria for GDM using a 2 h, 75 g oral glucose tolerance test (OGTT), are based upon risk of adverse pregnancy outcomes [4], which have been adopted by the World Health organisation (WHO) [5]. The recommended thresholds for diagnosing hyperglycaemia during pregnancy according to the IADPSG were defined at 1.75 times odds ratio (OR) for adverse outcomes, with fasting, one-hour and/or two-hour venous plasma glucose concentration cut offs of ≥ 5.1, ≥ 10.0 and/or ≥ 8.5 mmol/l respectively [4]. Adopting these new criteria, is anticipated to increase the GDM prevalence by 2–4 fold [4, 6, 7] with the additional associated costs. This has led to hesitation in implementing these new guidelines, especially if all pregnant women are to be offered an OGTT.

In 2015, the Swedish National Board of Health and Welfare (SNBHW) recommended the new IADPSG cut-off values for GDM but did not take a stand on GDM screening due to the limited knowledge base for outlining a screening program [8]. The new GDM diagnostic criteria will affect the test characteristics of current screening methods. Questions of whether the previously used screening criteria can still can be used or if an OGTT is required directly have been raised [8].

We have earlier published papers based on 3616 patients with an OGTT during pregnancy using traditional risk factors, random capillary and fasting blood glucose [9–11]. The capillary samples used whole blood glucose measurements, which were converted to venous plasma glucose values algorithmically [12], allowing a re-analysis of the value of risk factors and fasting venous plasma glucose for predicting GDM with the criteria based upon the new diagnostic criteria modified by the absence of the 1 h value (model I).

The primary aim of this study was to evaluate the test characteristics of different levels of fasting blood glucose (FBG) values, traditional risk factors alone and in combination with random blood glucose (RBG) as indications to perform an OGTT for diagnosing GDM based on the modified IADPSG criteria, in a Swedish, unselected population. The secondary aim was to evaluate the test characteristics of the same factors in relation to the HAPO data OR 2.0 (model II).

## Methods

This cross sectional study took place in Örebro County, Sweden, from 1 July 1994 to 30 June 1996. Details of the study design have been published earlier [9–11]. During this period all pregnant women (*n* = 4918) who attended maternal health care were offered a 75-g OGTT from gestational week 28–32, according to the WHO criteria at the time [13]. The women were instructed to intake carbohydrate rich food 2–3 days before the OGTT and fasting after 10 pm the day before the test. Capillary glucose samples were taken fasting and 2 h after the 75-g oral glucose load.

At the first maternal health visit the traditional risk factors (first-degree relative, obesity [≥90 kg, pre-pregnancy weight], previous large for gestational age (LGA) infant [≥4500 g or ≥mean + 2SD] or GDM) and maternal characteristics (age, parity and ethnic origin) were recorded.

Random blood glucose as measured four to six times during the pregnancy, starting at the end of first trimester with approximately 6 weeks intervals. If any of these RBG were≥ 9.0 mmol/l, an OGTT was carried out immediately. If this OGTT was negative in early pregnancy, before gestational week 28 (FBG < 6.7 mmol/l or 2 h-B-glucose < 9.0, the OGTT was repeated during gestational week 28–32, which was included in the study. One-hour glucose test was not available and was therefore not included in the GDM diagnosis.

The biochemical analysis of RBG, FBG and 2-h OGTT were analyzed using 5*μ*l capillary whole blood with Hemocue (Hemocue AB, Ängelholm, Sweden). The whole blood capillary values were converted to plasma venous values by multiplying by a constant factor of 1.11 [14] for fasting values and regarded as equivalent at 120 min [12]. Random whole blood capillary value was not converted.

Calculations were performed regarding; FBG, traditional risk factors and traditional risk factors in combination with RBG as screening test to predict GDM according to Model I (modified IADPSG criteria), which represent 1.75 OR for adverse outcomes in the HAPO study data: fasting ≥5.1 mmol/l and/or 2 h ≥ 8.5 mmol/l) [4], without the 1-h glucose value. Calculations for the same screening tests were performed predicting GDM according to Model II, 2.0 OR for adverse outcomes in the HAPO study data: fasting ≥5.3 mmol/l and/or 2 h ≥ 9.0 mmol/l [15]. Overt diabetes according to WHO is included in the different diagnostic groups.

The statistical analysis was performed using SPSS for Mac version 23.0 (SPSS inc., Chicago, IL, USA). Results are presented as mean ± SD or percentage with comparisons made using Mann-whitney U test or chi square test. Sensitivity, specificity, predictive values were calculated by using cross tabulations. Receiver operator characteristics (ROCs) of sensitivity plotted against 1-specificity was constructed for all the possible diagnostic predicted venous fasting plasma glucose (pvFPG) cut-off values and there AUC was calculated. Comparisons were made using 95% confidence intervals (95% CI).

## Results

During the study period 4918 women were offered an OGTT, 3616 (73.5%) eligible women accepted and were included in the study. Women foregoing the OGTT were older, less obese, more likely to be non-Nordic, but less likely to have past GDM or a family history of diabetes, prior GDM or a previous macrosomic baby as shown in Table 1 and as previously described [9–11]. The prevalence of GDM was 11.7% by modified IADPSG criteria (10.3% on fasting alone, 2.7% on 2 h alone and 1.3% with both values elevated) and 7.2% using model II criteria (6.4% on fasting alone, 1.6% on 2 h alone and 0.8% with both values elevated), 0.2% were diagnosed in early pregnancy using model I and II.

**Table 1:** Population characteristics of women who underwent OGTT and no OGTT, as previously reported [9–11]

Characteristics	OGTT (*n* = 3616)	No OGTT (*n* = 1302)	*P* value
Age (years) (SD)	27.9±4.8	28.5±5.0	0.005
Weight (Kg) (SD)	65.6±12.1	64.9±10.0	0.60
Length (cm) (SD)	166±6.0	166±6.4	0.53
BMI^a^ (kg/m^2^) (SD)	23.8±4.1	23.5±3.8	0.18
Non-Nordic origin (%)	11.2	14.3	0.001
Heredity (%)^b^	9.4	6.6	0.002
Obesity ≥ 90 kg (%)	4.5	2.6	0.003
BMI^a^ ≥ 30 kg/m^2^(%)	7.9	5.5	0.005
Prior infant ≥ 4500 g (%)	3.2	1.8	0.008
Prior GDM (%)	1.3	0.5	0.020
Primipara (%)	46	30.6	< 0.001

^a^*BMI* body mass index

^b^Heredity = family history of diabetes (first degree relative)

Results are presented as mean ± SD or percentage. Mann-whitney U test or chi square test.

Tables 2a and b show that risk factor screening alone or in combination with random capillary glucose showed low sensitivity using both model I (28, and 36% respectively) and model II (31 and 41% respectively). Specificities for model I was 86 and 84% respectively and 85 and 83% respectively for model II.

**Table 2:** Characteristics of risk factors and tests for detecting GDM defined as Model I^a^(A), modified IADPSG criteri and Model II^b^(B), HAPO data 2.0 OR criteria. 95% CI in parenthesis

Test pvFPG^c^ (mmol/l)	cFBG^d^ (mmol/l)	Occurrence *n* = 3616 (%)	Sensitivity (%)	Specificity (%)	PPV^e^ (%)	NPV^f^ (%)	AUC^g^ (%)
A.							
≥ 4.4	(4.0)	49	96 (94–98)	57 (55–59)	23 (21–25)	99 (98–99)	77 (75–78)
≥ 4.6	(4.1)	41	95 (93–97)	67 (65–68)	28 (25–30)	99 (99–99)	81 (79–83)
≥ 4.8	(4.3)	24	91 (88–94)	85 (83–86)	44 (41–48)	99 (98–99)	88 (86–90)
≥ 5.0	(4.5)	14	89 (86–92)	96 (95–96)	73 (69–77)	99 (98–99)	92 (91–94)
Traditional risk factors^h^		16	28 (24–32)	86 (84–87)	20 (17–24)	90 (89–91)	43 (40–46)
Traditional risk factors^h^ or RBG^i^ ≥8.0 (mmol/l)		19	36 (32–41)	84 (82–85)	23 (20–26)	91 (90–92)	40 (37–43)
B.							
≥ 4.4	(4.0)	49	96 (93–98)	54 (53–56)	14 (12–16)	99 (99–100)	75 (73–78)
≥ 4.6	(4.1)	41	96 (93–98)	64 (62–65)	17 (15–19)	100 (99–100)	80 (78–82)
≥ 4.8	(4.3)	24	93 (89–95)	81 (80–82)	28 (25–31)	99 (99–100)	87 (85–89)
≥ 5.0	(4.5)	14	91 (87–94)	92 (91–93)	46 (42–50)	99 (99–100)	91 (89–94)
≥ 5.2	(4.7)	8	89 (84–92)	98 (98–99)	78 (73–83)	99 (99–99)	94 (91–96)
Traditional risk factors^h^		16	31 (25–37)	85 (84–86)	14 (11–17)	94 (93–95)	42 (38–46)
Traditional risk factors^h^ or RBG^i^ ≥ 8.0 (mmol/l)		19	41 (35–47)	83 (82–84)	16 (13–19)	95 (94–96)	38 (34–42)

^a^Model I: Modified IADPSG criteria, 1.75 OR, equivalent cFBG ≥4.6 mmol/l or 2 h OGTT ≥8.5 mmol/l

^b^Model II: HAPO data 2.0 OR, equivalent cFBG ≥4.8 mmol/l or 2 h OGTT ≥9.0 mmol/l

^c^*pvFPG* Predicted venous fasting plasma glucose, equivalent cFBG value with conversion factor 1.11

^d^*cFBG* capillary fasting blood glucose

^e^*PPV* positive predictive value

^f^*NPV* negative predictive value

^g^*AUC* area under the curve

^h^Traditional risk factors = heredity (first-degree relative), obesity [≥ 90 kg, pre-pregnancy weight], previous LGA infant [≥ 4500 g or ≥ mean + 2SD], previous GDM

^i^*RBG* = random blood glucose

**Fig. 1:**
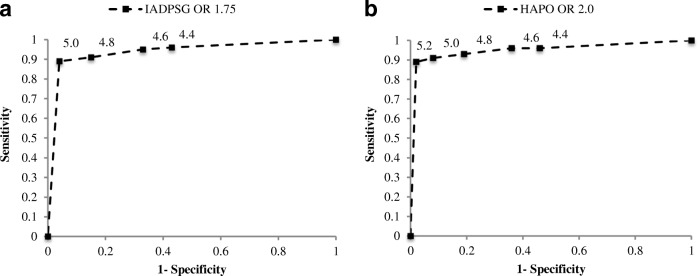
ROC curves for different predicted venous fasting plasma glucose cut-off values (mmol/l) as screening test for GDM according to modified IADPSG criteria (**a**) and HAPO data with OR 2.0 (**b**)

PvFPG cut-off values between 4.4 and 5.0 mmol/l had a sensitivity range between 89 and 96% and specificity 57–96% for the model I criteria. The optimal pvFPG cut- off value of 4.8 mmol/l occurred in 24% of the patient with 91% sensitivity, 85% specificity and 88% AUC. For model II, pvFPG cut-off values between 4.4 and 5.2 mmol/l had a sensitivity range between 89 and 96% and specificity 54–98%. The optimal pvFPG cut-off value of ≥5.0 mmol/l occurred in 14% of the patients with 91% sensitivity, 92% specificity, 91% AUC, see Fig. 1.

## Discussion

In this cross sectional, low-risk population based study, current screening methods for GDM screening in Sweden (traditional risk factors alone and/or combined with RBG) were found to be poorly predictive of GDM using different fasting and/or 2 h diagnostic thresholds based upon HAPO study 1.75/2.0 odds ratios for adverse pregnancy outcomes. Predicted venous plasma fasting glucose cut-off values of 4.8 and 5.0 mmol/l respectively were the optimal criteria for referral for an OGTT.

This study is based on a large, unselected, Swedish population that has a near 100% clinical attendance to maternal health care. Few women had an early diagnosis of GDM (with an OGTT following an elevated RBG), indicating minimal confounding by this local method for screening and supporting wider applicability of these results to areas that do not use RBG screening.

The main limitation is that the material was collected during 1994–1996, when capillary whole blood sampling was used in Sweden for OGTT. There is uncertainty about conversion factors from capillary blood to venous plasma [16]. A possible further limitation is that although all women were offered an OGTT, only 74% attended. Although participants had significantly higher rates of GDM risk factors than those declining an OGTT, suggesting a greater risk of GDM compared to the total population, the prevalence difference should not substantially affect the test characteristics in view of the high overall attendance.

Another limitation was that no one-hour glucose value was measured. 10. 4% of the HAPO cohort met the suggested IADPSG criteria for GDM when using fasting and 2-h values [4] compared to 11.7% in our study. In the HAPO study population 5.7% additional GDMs were identified by the 1 h values when using the IADPSG criteria. Assuming this, the prevalence of GDM in our study would be 17.4% compared to the HAPO study population of 17.8% [4], but with a different ethnic mix. The proportion of the population meeting the thresholds for the IADPSG criteria in the HAPO study (9.8%) was comparable to this study (10.3%) for fasting glucose but higher for the two-hour threshold (6.7% vs 2.7% respectively) [17]. Fewer women were diagnosed on the fasting glucose in HAPO compared with our study (55% vs 88% respectively). How much this was driven by the conversion of capillary whole blood glucose to venous plasma glucose for fasting and not the 2-h glucose is unclear. Our prevalence could also be marginally higher as women underwent OGTT from gestational week 28–32 instead of week 24–28 [18], and GDM does continue to develop after 28 weeks in approximately 5–16% of European women [19].

The rate of GDM in Sweden has been around 1–4% using the current screening and diagnostic criteria [10, 20–22]. This will increase markedly if an OGTT using IADPSG criteria is offered to all pregnant women [23]. This will be a challenge to the health care system, and underlines the need for further evaluating screening methods in relation to outcomes. While introducing the IADPSG criteria increases antenatal costs, it can reduce postnatal costs by a greater amount leading to net savings [24]. Reducing OGTTs where possible, is however, also preferable from the perspective of the women as they are time consuming and can be unpleasant.

Östlund et al. [11] studied the same population, but with different criteria, and reported higher sensitivity for traditional risk factors combined (48%) and in combination with RBG (69%) compared to the findings in our study. This could be expected with the higher Swedish criteria, which excluded many of those considered to have GDM under the IADPSG criteria. The present study shows that traditional risk factors for GDM have a low sensitivity with high amount of false negatives (large type II error) when applying the new criteria, making these methods unsuitable for clinical practice. These findings are consistent with a recent review article using different criteria [19]. They also concluded that although risk factors differ within and among countries there is no obvious “best” approach when using risk factors [25]. Implementing risk factor based screening can also be a challenge [26].

In the present study a pvFPG cut-off value of 4.8 and 5.0 mmol/l showed the best test characteristics with high sensitivity and specificity using both model I and II criteria. This could partly be due to that most GDM were diagnosed by the same fasting glucose used for the diagnosis, but is in accordance with Poomalar et al. They studied fasting plasma glucose as screening test in a population with 7.2% GDM and found that it is an effective screening tool. In their analysis a cut-off value of 4.7 mmol/l had the best test characteristics (sensitivity 88% and specificity 95%) [27].

Other studies using IADPSG criteria also showed FPG to be useful for simplifying the screening process and reducing the number of OGTTs [28–31] but with lower sensitivity in a low risk population [32]. Shen et al. reported in a large scale prospective cohort study using fasting, 1 and 2 h post load glucose, that a single fasting glucose measurement performs comparably to a 75-g OGTT in predicting risk of a LGA baby [33]. HAPO data showed that fasting, 1 h and 2 h glucose were highly predictive of cord- blood c-peptide values with fasting plasma glucose as the strongest predictor. Women with FPG positive IADPSG criteria had a higher prevalence of LGA (19.5%) compared to 1 and/or 2 h glucose [18].

The results in the present study, questions the validity of the current Swedish risk factor screening and RBG approach, since the methods are poorly predictive of GDM with the new IADPSG criteria. The low sensitivity results in around 70% of the GDM being missed using the new IADPSG criteria. Finding a model with a high sensitivity while avoiding many of the OGTTs would be of great clinical value.

A pvFPG of 4.8 and 5.0 mmol/l when using the model I and model II criteria would require 24 and 14% of women to progress to an OGTT respectively. As the sensitivity increases for fasting glucose values the specificity decreases. If the aim is to recognise disease the sensitivity could be prioritized before specificity.

Since the present analysis was based on conversion of capillary blood glucose to venous plasma sample there is a need of confirmation of our results. It would be valuable to study if a new screening model with new diagnostic criteria has an effect on the adverse pregnancy outcomes on a population-based level. We do not know if the IADPSG criteria are associated with less, later T2DM, and if the pregnancy complications such as LGA and caesarean section will be lowered across the population.

To address this, a large national stepped wedge randomized trial is running in Sweden, (started January 2018 (ISRCTN 41918550)). This study will evaluate old versus new diagnostic criteria for GDM in Sweden, and give more evidence for the association of different glucose levels in relation to pregnancy outcomes, health economics and long term effects on mother and offspring.

## Conclusion

In this cross sectional, low-risk population based study, current Swedish screening methods for GDM was found to be poorly predictive of GDM according to modified IADPSG criteria (OR 1.75) and HAPO data (OR 2.0). However, fasting glucose showed good test characteristics and could be an option for screening if resources for universal screening with OGTT are limited.

## Abbreviations

AUC: Area under the curve
BMI: Body mass index
CI: Confidence interval
FBG: Fasting blood glucose
GDM: Gestational diabetes mellitus
HAPO: Hyperglycaemia and adverse pregnancy outcomes
IADPSG: International Association of Diabetes and Pregnancy Study Group
LGA: Large for gestational age
OGTT: Oral glucose tolerance test
OR: Odds ratio
pvFPG: Predicted venous fasting plasma glucose
RBG: Random blood glucose
ROC: Receiver operating characteristic
SNBHW: Swedish National Board of Health and Welfare
T2DM: Type 2 diabetes mellitus
WHO: World Health Organization

## References

[1] Jiwani A, Marseille E, Lohse N, Damm P, Hod M, Kahn JG (2012). Gestational diabetes mellitus: results from a survey of country prevalence and practices. J. Matern. Fetal. Neonatal. Med..

[2] Macaulay S, Dunger DB, Norris SA (2014). Gestational diabetes mellitus in Africa: a systematic review. PLoS One.

[3] Hunt KJ, Schuller KL (2007). The increasing prevalence of diabetes in pregnancy. Obstet Gynecol Clin N Am.

[4] Consensus P, Metzger BE, Gabbe SG, Persson B, Buchanan TA, Catalano PA, International Association of Diabetes and Pregnancy Study Groups (2010). International association of diabetes and pregnancy study groups recommendations on the diagnosis and classification of hyperglycemia in pregnancy. Diabetes Care.

[5] Organization WH (2013). Diagnostic criteria and classification of hyperglycaemia first detected in pregnancy.

[6] Standards of Medical Care in Diabetes-2016 (2016). Summary of Revisions. Diabetes Care.

[7] Jenum AK, Morkrid K, Sletner L, Vangen S, Torper JL, Nakstad B (2012). Impact of ethnicity on gestational diabetes identified with the WHO and the modified International Association of Diabetes and Pregnancy Study Groups criteria: a population-based cohort study. European journal of endocrinology / European Federation of Endocrine Societies.

[8] Gränsvärden för graviditetsdiabetes (2015). stöd för beslut om behandling.

[9] Fadl H, Ostlund I, Nilsson K, Hanson U (2006). Fasting capillary glucose as a screening test for gestational diabetes mellitus. BJOG : an international journal of obstetrics and gynaecology.

[10] Östlund I, Hanson U (2003). Occurrence of gestational diabetes mellitus and the value of different screening indicators for the oral glucose tolerance test. Acta Obstet Gynecol Scand.

[11] Östlund I, Hanson U (2004). Repeated random blood glucose measurements as universal screening test for gestational diabetes mellitus. Acta Obstet Gynecol Scand.

[12] Alberti KG, Zimmet PZ (1998). Definition, diagnosis and classification of diabetes mellitus and its complications. Part 1: diagnosis and classification of diabetes mellitus provisional report of a WHO consultation. Diabetic medicine : a journal of the British Diabetic Association.

[13] WHO Expert Committee on Diabetes Mellitus (1980). second report. World Health Organ Tech Rep Ser.

[14] Fogh-Andersen N (2004). Evaluation of HemoCue glucose meter (201+): converting B-glucose to P-glucose. Point of Care.

[15] Coustan DR, Lowe LP, Metzger BE, Dyer AR (2010). The hyperglycemia and adverse pregnancy outcome (HAPO) study: paving the way for new diagnostic criteria for gestational diabetes mellitus. Am J Obstet Gynecol.

[16] Ignell C, Berntorp K (2011). Evaluation of the relationship between capillary and venous plasma glucose concentrations obtained by the HemoCue glucose 201+ system during an oral glucose tolerance test. Scand J Clin Lab Invest.

[17] Sacks DA, Hadden DR, Maresh M, Deerochanawong C, Dyer AR, Metzger BE (2012). Frequency of gestational diabetes mellitus at collaborating centers based on IADPSG consensus panel–recommended criteria. Diabetes Care.

[18] Group HSCR, Metzger BE, Lowe LP, Dyer AR, Trimble ER, Chaovarindr U, et al. Hyperglycemia and adverse pregnancy outcomes. N Engl J Med 2008;358(19):1991–2002. 10.1056/NEJMoa0707943 18463375

[19] Egan AM, Vellinga A, Harreiter J, Simmons D, Desoye G, Corcoy R (2017). Epidemiology of gestational diabetes mellitus according to IADPSG/WHO 2013 criteria among obese pregnant women in Europe. Diabetologia.

[20] Lindqvist M, Persson M, Lindkvist M, Mogren I (2014). No consensus on gestational diabetes mellitus screening regimes in Sweden: pregnancy outcomes in relation to different screening regimes 2011 to 2012, a cross-sectional study. BMC pregnancy and childbirth.

[21] Ignell C, Claesson R, Anderberg E, Berntorp K (2014). Trends in the prevalence of gestational diabetes mellitus in southern Sweden, 2003–2012. Acta Obstet Gynecol Scand.

[22] Anderberg E, Källén K, Berntorp K, Frid A, Åberg A (2007). A simplified oral glucose tolerance test in pregnancy: compliance and results. Acta Obstet Gynecol Scand.

[23] Simmons D (2016). Epidemiology of Diabetes in Pregnancy. Practical management of Diabetes in Pregnancy Second Edition ed.

[24] Duran A, Sáenz S, Torrejón MJ, Bordiú E, Del Valle L, Galindo M (2014). Introduction of IADPSG criteria for the screening and diagnosis of gestational diabetes mellitus results in improved pregnancy outcomes at a lower cost in a large cohort of pregnant women: the St. Carlos Gestational Diabetes Study Diabetes Care.

[25] Farrar D, Simmonds M, Bryant M, Lawlor DA, Dunne F, Tuffnell D (2017). Risk factor screening to identify women requiring oral glucose tolerance testing to diagnose gestational diabetes: a systematic review and meta-analysis and analysis of two pregnancy cohorts. PLoS One.

[26] Simmons D, Devers MC, Wolmarans L, Johnson E (2009). Difficulties in the use of risk factors to screen for gestational diabetes mellitus. Diabetes Care.

[27] Poomalar G, Rangaswamy V (2013). A comparison of fasting plasma glucose and glucose challenge test for screening of gestational diabetes mellitus. J Obstet Gynaecol.

[28] Mahdavian M, Hivert M-F, Baillargeon J-P, Menard J, Ouellet A, Ardilouze J-L (2010). Gestational diabetes mellitus: simplifying the international association of diabetes and pregnancy diagnostic algorithm using fasting plasma glucose. Diabetes Care.

[29] Agarwal MM, Weigl B, Hod M (2011). Gestational diabetes screening: the low-cost algorithm. Int J Gynecol Obstet.

[30] Agarwal MM, Dhatt GS, Shah SM (2010). Gestational diabetes mellitus: simplifying the international association of diabetes and pregnancy diagnostic algorithm using fasting plasma glucose. Diabetes Care.

[31] Anderson V, Ye C, Sermer M, Connelly PW, Hanley AJ, Zinman B (2013). Fasting capillary glucose as a screening test for ruling out gestational diabetes mellitus. J Obstet Gynaecol Can.

[32] Ryser Ruetschi J, Jornayvaz FR, Rivest R, Huhn EA, Irion O, Boulvain M (2016). Fasting glycaemia to simplify screening for gestational diabetes. BJOG.

[33] Shen S, Lu J, Zhang L, He J, Li W, Chen N (2017). Single fasting plasma glucose versus 75-g oral glucose-tolerance test in prediction of adverse perinatal outcomes: a cohort study. EBioMedicine.

